# Clinical factors associated with peripherally inserted central catheters (PICC) related bloodstream infections: a single centre retrospective cohort

**DOI:** 10.1186/s13756-023-01209-z

**Published:** 2023-01-30

**Authors:** Koko Barrigah-Benissan, Jerome Ory, Claire Simon, Paul Loubet, Aurelie Martin, Jean-Paul Beregi, Jean-Philippe Lavigne, Albert Sotto, Romaric Larcher

**Affiliations:** 1 Department of Microbiology and Infection Control, CHU Nimes, Nimes, France; 2 Bacterial Virulence and Chronic Infections, INSERM U1047, University of Montpellier, Montpellier, France; 3 Department of Pharmacy, CHU Nimes, Nimes, France; 4 Department of Infectious and Tropical Diseases, CHU de Nimes, 1 Place Robert Debré, 30000 Nimes, France; 5 Department of Medical Imaging, CHU Nimes, Nimes, France; 6 PhyMedExp, INSERM, CNRS, University of Montpellier, Montpellier, France

**Keywords:** PICC, CR-BSI, Fever, Chills, PICC maintenance duration

## Abstract

**Background:**

Despite their spread in daily practice, few data is available on clinical factors associated with peripherally inserted central catheter (PICC)-related bloodstream infections (PR-BSI). We aimed to assess the PR-BSI incidence, microbiology, and factors associated with PR-BSI with a focus on clinical symptoms.

**Methods:**

We conducted a retrospective cohort study in a French university hospital. We screened all PICC insertions performed from April 1st, 2018, to April 1st, 2019, and included PICC insertions in adult patients. We assessed the PR-BSI incidence, the factors associated with PR-BSI using a Cox model, and negative and positive predictive values (NPVs and PPVs) of each clinical sign for PR-BSI.

**Results:**

Of the 901 PICCs inserted in 783 patients (38,320 catheters days), 214 PICCs (24%) presented with a complication. The most prevalent complication was PR-BSI (1.9 per 1000 catheter days; 8.1% of inserted PICCs ). Enterobacterales (*N* = 27, 37%) and coagulase negative Staphylococci (*N* = 24, 33%), were the main microorganisms responsible for PR-BSI. Factors independently associated with occurrence of PR-BSI were fever (hazard ratio 13.21, 95% confidence interval 6.00–29.11, *p* < 0.001) and chills (HR 3.66, 95%CI 1.92–6.99, *p* < 0.001). All clinical signs and a duration of PICC maintenance ≥ 28 days, had a low PPVs (≤ 67.1%) but high NPVs (≥ 92.5%) for PR-BSI.

**Conclusions:**

Monitoring of clinical signs, especially fever and chills, with caution and limitation of device maintenance duration, could improve PICC management.

## Background

Since their introduction in late 1970s [1], peripherally inserted central catheters (PICC, also known as PICC-line) widely spread in daily practice. They are indicated for intermediate-term venous access (7 days to 6 months) for some concrete indications as irritant or vesicant infusions (total parenteral nutrition or chemotherapy), difficult venous access and prolonged antimicrobial therapies [2]. PICC insertion occurs through a peripheral upper arm vein, avoiding iatrogenic complications and ensuring a safe and easy removal compared to other central venous catheters (CVCs) which they partially replaced [3]. Despite some advantages, mechanical complications as occlusion, accidental withdrawal [4], deep venous thrombosis [5], and catheter-related bloodstream infections (CR-BSIs) [6, 7] were reported during PICC use. Rates of PICC-related bloodstream infections (PR-BSIs) compared to other CVCs infections has been a controversial and disputed subject. Now PR-BSI rates are recognized similar to those of conventional CVCs [8, 9]. Nonetheless, in a recent national public health survey in France, one quarter of the 30 million of catheters implanted every year presented with a CR-BSI. Among these infections, 16.8% were related to a PICC, making PR-BSI the most important and preventable PICC-related complication [10–12].

Whilst abundant literature is available on PICC related complications, PR-BSI risk factors are still poorly assessed [13]. Published data remains scarce [9, 13–18], particularly for medical ward inpatients and outpatients, since most of the data focuses on intensive care unit (ICU) [19].

A study aiming to assess PICC-related complication incidence, with a highlight on PR-BSI, to describe the microorganisms involved and to assess risk factors associated with PR-BSI with a focus on clinical signs, is lacking.

## Methods

### Study aim, design and settings

This study aims to assess PICC-related complication incidence, particularly the PR-BSI incidence and to describe the microorganisms involved and risk factors associated with PR-BSI with a focus on clinical signs in patients from a tertiary hospital.

We conducted a retrospective, single centre, observational cohort study in Nimes University Hospital, from April 1st, 2018, to April 1st, 2019. In the medical imaging department of this 2094-beds University Hospital, single or double lumen PICC (Bard Access Systems, Salt Lake City, UT, USA) ultrasonography guided insertions are performed on inpatients and outpatients 5 days a week. Each PICC insertion request is forwarded to, registered, and validated or not by the Pharmacy Department. PICC insertion is performed in aseptic conditions according to the French Society of Infection Control (SF2H) guidelines [20]. After insertion, PICC position is verified with a chest X-ray and adjusted if required. Normal saline is used for preventing lumen occlusion and maintaining PICC patency, as recommended by national and international guidelines [2, 20].

### Patients

We screened all consecutive patients with at least one PICC placement between April 1st, 2018, and April 1st, 2019, using the pharmacy registry of PICC insertion requests. All adult patients were included in the study. When a patient had more than one PICC placement, all PICC placements were considered. Patients under 18-year-old and those lost to follow-up between PICC insertion and PICC removal were excluded.

### Data collection

Patient’s demographic, clinical and biological data were collected from the hospital electronic medical record. Age, sex, weight, height, body mass index (BMI), medical history, reason for PICC placement, insertion date, removal date, type of PICC, site of insertion, reason for PICC removal, ongoing treatment (especially corticostoreids or other immunosuppressive treatments) and vital status at PICC removal were collected. When a bloodstream sample was diagnosed positive by the microbiology laboratory, more information was collected on the laboratory software (number of samples collected, number of positive samples, central and peripheral blood culture results, insertion site culture and catheter culture results, lag time between central and peripheral positive blood cultures, microorganism identification and antimicrobial resistance). Patients’ Charlson comorbidity index was calculated [21]. Microorganisms resistance diagnosis were based on the European Committee on Antimicrobial Susceptibility Testing (EUCAST) guidelines [22]. An adjudication committee, made up of an infection control specialist (J.O.), an infectious disease physician (A.S) and an intensivist (R.L.), analysed the medical records to ensure the diagnosis met definition criteria of PICC-related infections (PRI) [23]. For each case of BSI, alternative sources of infection were carefully checked by the adjudication committee by reviewing the patient chart and all microbiological culture results. In case of discrepancy, diagnosis was discussed between the committee members until a consensus was reached.

### Definitions

We defined PRI according to the French Intensive Care Society (SRLF) guidelines [23], which are in line with the Centers for Disease Control and Prevention (CDC) and the European Centre for Disease Control and Prevention (ECDC) guidelines [24–26].

We defined PICC colonization as a quantitative catheter culture ≥ 10^3^ CFU/mL (according to Brun-Buisson) without bacteraemia or clinical signs [23].

We defined non-bacteraemia PRI (NB-PRI), in the absence of bacteraemia, as a combination of: (i) a quantitative catheter culture ≥ 10^3^ CFU/mL and (ii) (a) signs of local infection (purulent discharge from the PICC insertion site or tunnel infection); and/or (b) systemic signs, with complete or partial resolution of systemic signs of infection within 48 h after PICC removal [23].

We defined PR-BSI as an association of: (i) the occurrence of either bacteraemia or fungaemia during the 48-h period surrounding catheter removal (or a suspected diagnosis of PRI when the PICC is not removed immediately); (ii) and either a positive culture with the same microorganism on one of the following samples: insertion site culture, or catheter culture ≥ 10^3^ CFU/mL or positive central and peripheral blood cultures with the same microorganism, with a central/peripheral positive blood culture lag time > 2 h, with central blood cultures being positive earlier than the peripheral ones [23].

### Statistical analysis

PICC insertion was the unit for statistical analyses. Data are described as median and interquartile range (IQR) or number and percentage as appropriate. We assessed factors associated with PR-BSI using a Cox model. Factors with a *p*-value ≤ 0.1 in the univariate analysis were included in the multivariable analysis. Results of the Cox model were reported as hazard ratio (HR) with 95% confidence interval (95% CI). We plotted receiver operating characteristic (ROC) curves for clinical signs associated with PR-BSI and for PICC maintenance duration. We calculated the negative and positive predictive values (NPVs, PPVs) for each clinical sign, and identified the optimal cut-off value of catheter duration for PR-BSI occurrence by maximizing the Youden index. All tests were two-sided and *p*-values less than 0.05 were considered statistically significant. We performed all analyses using *R* software, version 4.2.0 (The *R* Foundation for Statistical Computing, Vienna, Austria).

## Results

**Fig. 1:**
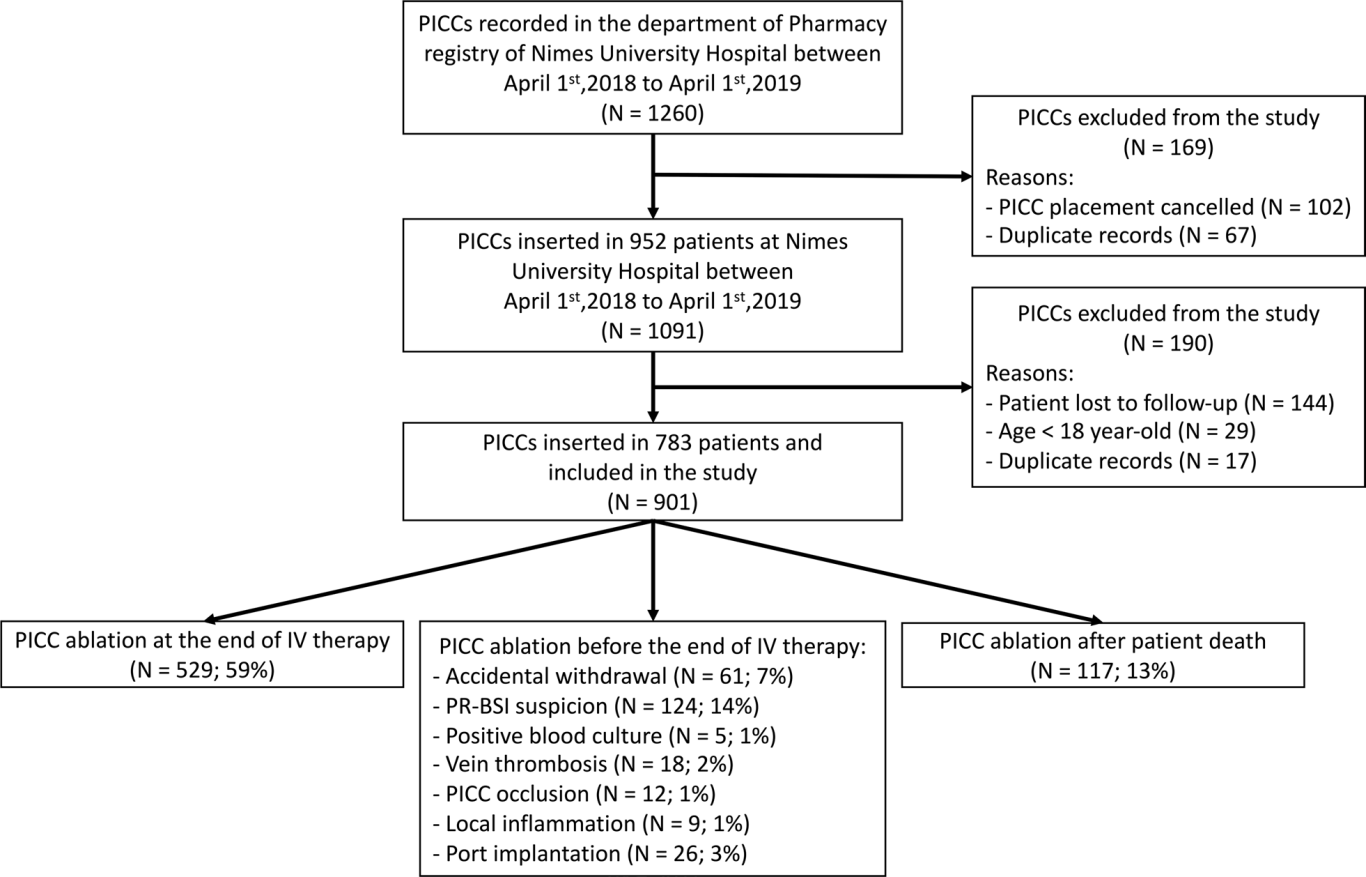
Flow chart of peripherally inserted central catheters (PICC) placement

From April 1st,2018 to April 1st,2019; amongst 1091 PICCs inserted in 952 patients, 901 PICCs inserted in 783 patients met inclusion criteria and were included in the analysis (Fig. 1). The median follow-up was 21 days (IQR, 9–43). More than a half of PICCs were removed at the end of intravenous therapy (*N* = 529; 59%), 38% (*N* = 346) because of a complication and 3% (*N* = 26) after port implantation. The median time for PR-BSI occurrence was 30 (IQR, 16–76).

Characteristics of the study population are presented in Table 1. The median age for patients with a PICC insertion was 70.9 years (IQR, 59.4–79.8). The median BMI was 24.5 kg/m^2^ (IQR, 21.2–28.9). More than a half of patients had a peripheral vascular disease, a quarter had a chronic heart failure and 22% a metastatic cancer. The median Charlson comorbidity index was 6 (IQR, 3–9).

**Table 1:** Characteristics of the study population and peripherally inserted central catheters (PICC)

	Total (*N* = 901) *N* (%) or median (IQR)	No PR-BSI (*N* = 828) N (%) or median (IQR)	PR-BSI (*N* = 73) *N* (%) or median (IQR)
Patients, N	783	713	70
Sex ratio M/F	500/401	461/367	37/33
Age (year)	70.9 (IQR, 59.4–79.8)	71.0 (IQR, 59.8–80.5)	69.8 (IQR, 58.8–74.1)
Body Mass Index (kg/m^2^)	24.5 (IQR, 21.2–28.9)	23.8 (IQR, 20.7–28.1)	24.4 (IQR, 21.1–28.7)
Charlson comorbidity index	6 [3–9]	6 [3–8]	7 [4–10]
*Main comorbidities*			
Corticosteroids	141 (18%)	112 (16%)	29 (41%)
Immunosuppressors	101 (13%)	78 (11%)	23 (33%)
Solid tumour	142 (18%)	129 (18%)	13 (19%)
Metastatic solid tumour	178 (23%)	151 (21%)	27 (38%)
Haematological malignancy	69 (9%)	51 (7%)	18 (26%)
Leukaemia	46 (6%)	36 (5%)	10 (14%)
Lymphoma	23 (3%)	15 (2%)	8 (11%)
*PICC lumen*			
1 lumen	712 (79%)	669 (81%)	43 (59%)
2 lumens	189 (21%)	159 (19%)	30 (41%)
*Reason for PICC insertion*			
Antimicrobial therapy	408 (45%)	397 (48%)	11 (15%)
Chemotherapy	240 (27%)	201 (24%)	39 (53%)
Difficult venous access	154 (17%)	143 (17%)	11 (15%)
Total parenteral nutrition	52 (6%)	44 (5%)	8 (11%)
Iterative venous access	47 (5%)	43 (5%)	4 (5%)
*Insertion site*			
Left basilic vein	470 (52%)	436 (53%)	34 (47%)
Right basilic vein	135 (15%)	124 (15%)	11 (15%)
Left brachial vein	209 (24%)	189 (22%)	21 (29%)
Right brachial vein	72 (8%)	67 (8%)	5 (7%)
Left cephalic vein	12 (1%)	10 (1%)	2 (3%)
Right cephalic	1 (< 1%)	1 (< 1%)	0 (0%)
Femoral	1 (< 1%)	1 (< 1%)	0 (0%)
PICC maintenance (days)	21 (IQR, 9–43﻿]	20 (IQR, 9–41]	30 (IQR, 16–76)
*Reason for PICC removal*			
End of IV therapy	529 (59%)	529 (64%)	0 (0%)
Accidental withdrawal	61 (7%)	60 (7%)	1 (1%)
PR-BSI suspicion	124 (14%)	62 (7%)	62 (85%)
Positive blood culture	5 (1%)	2 (<1%)	3 (4%)
Vein thrombosis	18 (2%)	17 (2%)	1 (<1%)
PICC occlusion	12 (1%)	11 (1%)	1 (<1%)
Local inflammation	9 (1%)	8 (1%)	1 (<1%)
Port implantation	26 (3%)	25 (3%)	1 (<1%)
Death	117 (13%)	114 (14%)	3 (4%)

*BSI* Bloodstream infection, *COPD* Chronic obstructive pulmonary disease, *IQR* Interquartile range, *PR-BSI* PICC related bloodstream infection, *IV* Intravenous

Antimicrobial therapy (*N* = 408; 45%); chemotherapy (*N* = 240; 27%) and difficult venous access (*N* = 154; 17%) were the main reasons for PICC insertion. Inserted PICCs had mostly one lumen (*N* = 712; 79%) and were inserted in the left basilic or brachial veins (*N* = 470; 52%, or *N* = 209; 24%). The median PICC maintenance duration was 21 days (IQR, 9–43) accounting for 38,320 catheter days, and PICCs were essentially removed at the end of IV therapy (*N* = 529; 59%). However, an accidental withdrawal before the end of therapy occurred in 69 cases (7%), and PICCs were also removed immediately in case of CR-BSI suspicion (*N* = 124; 14%) or death (*N* = 117; 13%). Characteristics of PICCs are summarized in Table 1.

Around one quarter of PICCs (*N* = 214, 24%) presented with a complication (Table 2). The first complication encountered was PR-BSI (*N* = 73, 8.1%, 1.9 per 1000 catheter days). Accidental withdrawal (*N* = 61, 6.8%, 1.6 per 1000 catheter days), vein thrombosis (*N* = 14, 1.6% and 0.4 per 1000 catheter days), catheter occlusion (*N* = 12, 1.3%, 0.3 per 1000 catheter days), local signs of inflammation (*N* = 4, 0.4%, 0.1 per 1000 catheter days) and NB-PRI (*N* = 3, 0.4%, < 0.1 per 1000 catheter days) were less frequent.

**Table 2:** Type, rate and incidence of peripherally inserted central catheters (PICC) complications

Complications	Number of PICC	Rates (%)	Incidence (per 1000 catheter days)
PR-BSI	73	8.1	1.9
Accidental withdrawal	61	6.8	1.6
PICC colonization	47	5.2	1.2
Vein thrombosis	14	1.6	0.4
Catheter occlusion	12	1.3	0.3
Local inflammation	4	0.4	0.1
NB-PRI	3	0.3	< 0.1

*PR-BSI* PICC related bloodstream infection, *NB-PRI* Non bacteraemia PICC related infection

Among the microorganisms involved in PR-BSI, Enterobacterales were the main species (*N* = 27, 37%), followed by Coagulase Negative Staphylococci (CoNS) (*N* = 24; 33%), *Staphylococcus aureus* (*N* = 7; 10%), *Candida* species (*N* = 7; 10%) and non-fermenting Gram-Negative Bacilli (*N* = 5; 7%). Polymicrobial CR-BSI with two species accounted for 19 cases (26%) and 5 cases (7%) were documented with more than 2 species. Moreover, 47 PICCs presented with a colonization (6.8%, 1.2 per 1000 catheter days), predominantly due to CoNS (*N* = 33, 70%) and Enterobacterales (*N* = 6, 13%).

Characteristics of the species involved in infectious complications are listed in Table 3.

**Table 3:** Species involved in infectious complications of peripherally inserted central catheter (PICC)

Species	PR-BSI (N, %)	PICC colonization (*N*, %)	Total (*N*, %)
*Gram positive cocci*			
Coagulase Negative Staphylococci	24 (33%)	33 (70%)	57 (48%)
Linezolid resistant	2 (3%)	0 (0%)	2 (2%)
*Staphylococcus aureus*	7 (10%)	1 (2%)	8 (7%)
MRSA	5 (7%)	0 (0%)	5 (4%)
*Enterococcus spp*	2 (3%)	1 (2%)	3 (2.5%)
*Streptococcus spp*	1 (1%)	0 (0%)	1 (1%)
*Gram negative bacilli*			
Enterobacterales	27 (37%)	6 (13%)	33 (28%)
3GCR	28 (38%)	0 (0%)	28 (23%)
ESBL	10 (14%)	0 (0%)	10 (8%)
AMPC	16 (23%)	0 (0%)	16 (13%)
CRE	7 (10%)	0 (0%)	7 (6%)
Non-fermenters	5 (7%)	3 (6%)	8 (7%)
Pip-Taz resistant *P. aeruginosa*	2 (3%)	0 (0%)	2 (2%)
Ceftazidime resistant *P. aeruginosa*	1 (1%)	0 (0%)	1 (1%)
CR *P. aeruginosa*	1 (1%)	0 (0%)	1 (1%)
*Candida spp.*	7 (10%)	3 (6%)	11 (9%)
Total	73	47	120

*CR* Carbapenem resistant, *CRE* Carbapenem resistant Enterobacterales, *ESBL* Extended spectrum beta-lactamase, *MRSA* Methicillin resistant *Staphylococcus aureus*, *Pip-Taz* Piperacillin-tazobactam, *PR-BSI* PICC related bloodstream infection, *3GCR* 3^rd^ generation cephalosporin resistance

**Fig. 2:**
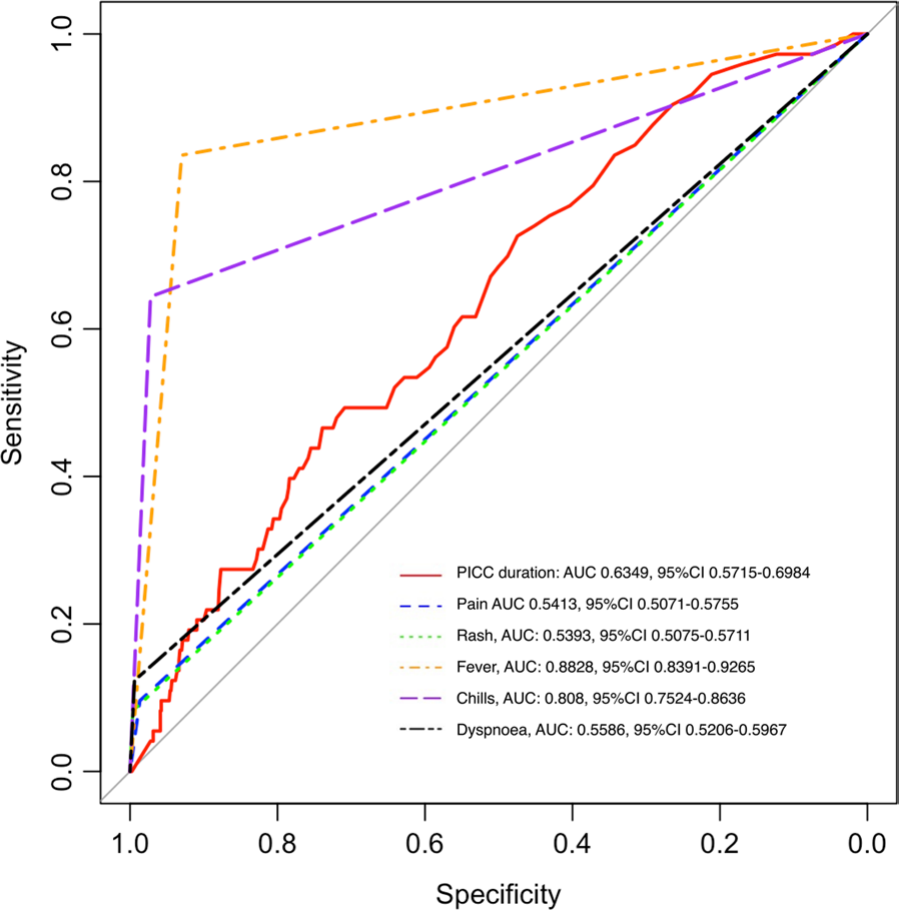
Receiver operating characteristics (ROC) curves for peripherally inserted central catheter (PICC)-associated bloodstream infection on the basis of the number of catheter days (red line) and clinical signs such as pain (blue dashed line), rash at the PICC insertion site (green pointed line), fever (orange dashed line), chills (purple dashed line), dyspnea (black dashed line). Area under the ROC curves (AUCs) are given with the 95% confidence interval (95% CI)

Fever and chills were the most common clinical signs in 84% and 64% of patients with PR-BSI, respectively. These two clinical signs showed the best prediction capacity for PR-BSI: area under the ROC curve (AUC) 0.8828, 95%CI 0.8391–0.9265 for fever and 0.808, 95%CI 0.7524–0.8636 for chills (Fig. 2). Importantly, all clinical signs, namely pain or rash at PICC insertion site, fever, chills, or dyspnoea had high specificity but low sensitivity for PR-BSI detection (Table 4). Accordingly, the NPVs of clinical signs were satisfactory, especially for fever (98.5%) and chills (96.9%), whereas the PPV were low. The higher PPV were 66.7% for the occurrence of a rash at PICC insertion site and 67.1% for the occurrence of chills (Table 4).

**Table 4:** Factors independently associated with peripherally inserted central catheters (PICC) related bloodstream infection (PR-BSI), and predictive values of clinical signs for PR-BSI

	Multivariate analysis			Diagnostic ability of clinical signs			
Variables	Hazard ratio	95% CI	*p*-value	Sensitivity	Specificity	PPV	NPV
*Patient conditions*							
Immunosuppr. therapy^1^	1.07	0.80–1.43	0.63	–	–	–	–
Malignancy^2^	1.08	0.67–1.75	0.75	–	–	–	–
Dementia	1.62	0.18–14.58	0.67	–	–	–	–
Clinical signs							
Local signs							
Pain	0.55	0.17–1.81	0.33	9.6%	98.7%	38.8%	92.5%
Rash	2.82	0.82–9.69	0.10	8.2%	99.6%	66.7%	92.5%
*Systemic signs*							
Fever	13.21	6.00–29.11	< 0.001	83.6%	93.0%	51.3%	98.5%
Chills	3.66	1.92–6.99	< 0.001	64.4%	97.2%	67.1%	96.9%
Dyspnoea	1.24	0.53–2.94	0.62	12.0%	99.4%	64.3%	93.0%
PICC duration ≥ 28 days	–	–	–	53.4%	62.8%	11.2%	93.9%

^1^at least one of the following: corticosteroids, azathioprine, mycophenolate mophetil, tacrolimus, ciclosporin. ^2^solid tumour or haematological malignancy. *PPV* Positive predictive Value, *NPV* Negative Predictive Value, 95% CI 95%confidence interval

**Fig. 3:**
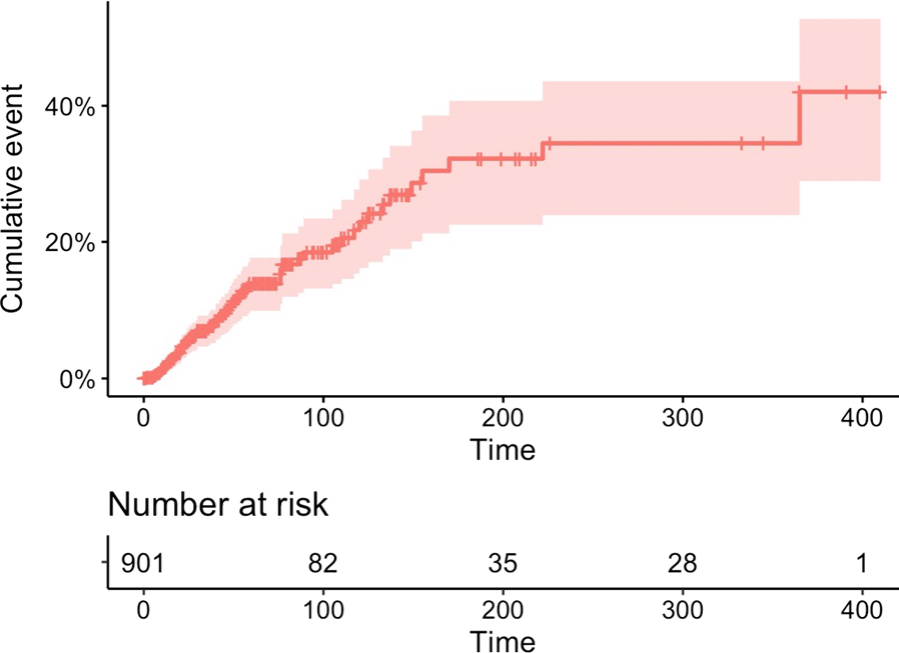
Incidence of peripherally inserted central catheters (PICC) related bloodstream infection during PICC maintenance (in days) with 95% confidence interval

As illustrated in the Fig. 3, the risk of PR-BSI increased with the duration of PICC maintenance. The risk increased mainly during the first 6 months with a probability of PR-BSI at 32.2% (95%CI 22.5–40.7) at day-180. The AUC of PICC maintenance duration for PR-BSI development was 0.6349, 95%CI 0.5715–0.6984 and the optimal cut-off value of catheter day associated with PR-BSI development was 28 days. Similar to clinical signs, the PPV for PR-BSI development of a PICC maintenance duration ≥ 28 days was very low at 11.2% whereas the NPV was at 93.9%.

In the univariate analysis the variables associated with occurrence of PR-BSI were: an history of immunosuppressive treatments (either corticosteroids or immunosuppressors, or both; HR 1.79, 95%CI 1.38–2.32, *p* < 0.001), an history of malignancy (solid tumour or haematological malignancy; HR 1.68, 95%CI 1.2–2.36, *p* = 0.003), dementia (HR 8.25, 95%CI 1.12–61, *p* = 0.04), insertion of a double-lumen PICC (HR 2.24, 95%CI 1.41–3.58, *p* < 0.001) and occurrence of clinical signs: pain at insertion site (HR 6.65, 95%CI 3.01–14.7, *p* < 0.001), rash at insertion site (HR 10.2, 95%CI 4.36–23.8, *p* < 0.001), fever (HR 30.9, 95%CI 16.5–57.7, *p* < 0.001), chills (HR 25.6, 95%CI 15.2–43, *p* < 0.001).

In the multivariable analysis only fever (HR 13.21, 95%CI 6.00–29.11, *p* < 0.001) and chills (HR 3.66, 95%CI 1.92–6.99, *p* < 0.001) were independently associated with the occurrence of PR-BSI (Table 4).

## Discussion

This retrospective cohort study reports the results of 901 PICC insertions in 783 patients accounting for 38,320 catheters days. 214 PICCs (24%) presented with a complication, mainly a PR-BSI (*N* = 73, 8.1%) with a 1.9 per 1000 catheter days incidence rate. Enterobacterales (*N* = 27, 37%) and CoNS (*N* = 24, 33%) were the main microorganisms involved in PR-BSI. Moreover, we highlighted the importance of clinical signs fo PR-BSI monitoring, reporting fever and chills as factors independently associated with PR-BSI occurrence.

Over the last decade, PICC use increased in hospitals [27], particularly in university hospitals [10]. PR-BSIs also increased at the same time [10, 28, 29]. One important finding of this study was the 1.9 per 1000 catheter days incidence rate of PR-BSI, which was in line with the median 2.1 per 1000 catheter days incidence rate reported in meta-analyses of international studies [9, 30, 31]. Previous studies found various incidence rates ranging from 0.6 to 3.3 per 1000 catheter days [4, 16–18, 32]. Importantly, differences in PR-BSI incidence may be related to the time period of the studies and differences in catheter-related infection definitions across these studies [16]. Moreover, several definitions used in numerous studies were reported unsuitable for research [33]. Nonetheless, we must acknowledge the PR-BSI incidence rate found in this study was above the 1 per 1000 catheter days threshold target for CR-BSI prevention intervention suggested by infection control experts [12]. In this global context, CVCs and particularly PICC were pointed as priorities for infection prevention measures [28]. Multimodal process for better CR-BSI prevention and control including practice change based on knowledge, education, and behavioural interventions in our hospital are encouraged [12]. Recent guidelines [34] suggested, practical healthcare workers team education and training, promoted by a multidisciplinary team with checklists, continuous improvement programs and bundles implementation [35].

Another significant finding of this work was the importance of clinical monitoring of PR-BSI. Indeed, this study identified a strong correlation between clinical signs such as fever and chills, and PR-BSI. Most importantly, clinical signs showed high specificity and NPV but unsatisfactory sensibility and PPV. The absence of clinicals signs is therefore significantly indicative of the absence of PR-BSI as suggested by others in ICU settings [36]. Amongst clinical signs, fever and chills have the best NPVs. Fever was previously reported as indicative of CR-BSI along with other infection signs [37, 38]. In line with previous studies [39–41], we reported that prolonged duration of catheterization increased the risk for PR-BSI (see supplementary materials). However, our work underlined the poor ability of PICC maintenance duration to predict PR-BSI. Indeed, the threshold value of 28 catheter days was associated with very low PPV but satisfactory NPV for PR-BSI, as previously reported [42]. These results suggest a close clinical signs monitoring could rapidly help diagnose and treat PR-BSI particularly in patient with a prolonged PICC dwelling time. These results also supports the recent recommendation of catheter duration limitation to the shortest requested to limit PR-BSI [34]. Available data also suggests that clinicians should limit the number of catheter lumen [9, 14, 43] and concurrent catheter [34].

Immunocompromised patients, particularly those treated with immunosuppressive treatments and/or with metastatic cancer have been identified at risk for PR-BSI. Immunosuppression, particularly neutropenia, was previously reported as risk factor for PR-BSI [40, 44–47], prompting clinicians to monitor PICC in immunocompromised patients with caution. Surprisingly, those patients did not seem at higher risk for PR-BSI in our study. During the study, a high nurse-to-patient ratio in haematology and oncology wards, local guidelines, and specific courses on PICC use for nurses were available in our institution. This may have impacted the quality of PICC care which has been widely reported to decrease PR-BSI incidence rate [34].

Previous studies on PR-BSI microbiology reported CoNS as the predominant microorganisms [11, 13, 39, 48]. Nonetheless, recent evidences [48–51] suggested a change in this trend with the rising of Gram negative bacilli as main microorganisms associated with PR-BSI. With a majority of PR-BSI related to Enterobacterales (37%), the results of our study tend to support the later. Yet, CoNS are still significantly associated with PR-BSI (33%) and responsible for most of PICC colonisations. The large proportion of immunocompromised patients in our cohort (almost a half of patients had a haematological malignancy or a cancer, and more than two thirds of patients with a PR-BSI), may explain the high prevalence of Enterobacterales associated PR-BSIs since these patients are most at risk to be infected with their own Enterobacterales [52]. Another possible explanation for these results was the inclusion of patients in home-hospitalization. This finding has important implications for the implementation of infection control bundle in PR-BSI prevention, especially regarding hand disinfection and skin antisepsis during PICC care and dressing management [12, 35].

This study also reported low rates of PICC related complications other than PR-BSI. We found an accidental withdrawal incidence rate of 1.6 per 1000 catheter days (*N* = 61, 6.8%), lower than those reported by Valbousquet et al*.* (2.8 per 1000 catheter days) [17] and Grau et al*.* 8.0% [16]. However, others  found lower rates at 2.4% [4] and 5% [18], respectively, but reported rates instead of incidence which limited comparison with our results. Importantly, accidental withdrawal was identified as a common complication in PICC use especially in patients older than 70 year-old, which is the median age in our study population [16, 53]. We reported a vein thrombosis incidence rate at 0.4 per 1000 catheter days, also lower than those previously reported [19, 53, 54]. PICCs are described as more thrombogenic than CVCs [19, 55], particularly in cephalic vein position [56]. Consequently, PICC were mainly inserted in basilic, or brachial vein as recommended in our local protocol. In addition, more than a third (27%) of the patients received an anticoagulant therapy. This may have limited the incidence rate of thrombosis in our cohort.

This study has several limitations. First, the single centre design of the study could limit extrapolation of the results. Second, the relatively small size of our cohort limited the weight of some factors such as BMI > 40 kg/m^2^, number of lumen or total parenteral nutrition which are recognized at risk factors for CR-BSI [40, 57]. However, to the best of our knowledge, this study is the first to analyse the association between clinical signs and PR-BSI. Third, the retrospective design of the study limits our analyses to available data in medical records and may induce bias in data collection and results interpretation. Some risk factors such as the microbial colonization at the catheter hub and at insertion site, or the outpatient/inpatient status, could not be assessed. However, combining medical, microbiological, and administrative data, added to the adjudication committee for PR-BSI diagnosis in accordance with international expert consensus tends to limit this bias.

## Conclusions

Complications occurred in 24% of PICC, and PR-BSI was the most prevalent one with a 1.9 per 1000 catheter days incidence rate. PR-BSIs were mainly caused by Enterobacterales and CoNS. Clinical signs and PICC maintenance duration ≥ 28 catheter days, had better NPVs than PPVs for PR-BSI diagnosis. Fever and chills had the best NPV and were independently associated with PR-BSI occurrence.

These results suggest that health workers should cautiously monitor PICC insertion site, and especially fever and chills, and limit the duration of PICC maintenance to the minimum required. They also prompt patients to self-monitoring. Further studies are mandatory to assess whether improving patients and healthcare workers education on PICC management through the development of an infection prevention bundle and continuous evaluation could reduce PR-BSI under 1 per 1000 catheter days.

## Data Availability

The authors consent to share the collected data with others. The raw data supporting the conclusions of this article will be made available by the authors, without undue reservation. Data will be available immediately after the main publication and indefinitely.

## Abbreviations

AUC: Area under the ROC curve
BMI: Body mass index
CDC: Centers for disease control and prevention
CoNS: Coagulase negative staphylococci
CR: Carbapenem resistant
CRE: Carbapenem resistant enterobacterales
CR-BSI: Catheter-related bloodstream infections
CVC: Central venous catheters
ECDC: European centre for disease control and prevention
ESBL: Extended spectrum beta-lactamase
EUCAST: European committee on antimicrobial susceptibility testing
HR: Hazard ratio
IQR: Interquartile range
MRSA: Methicillin resistant *Staphylococcus aureus*
NB-PRI: Non-bacteraemia PRI
NPV: Negative predictive value
PICC: Peripherally inserted central catheter
Pip-Taz: Piperacillin-tazobactam
PPV: Positive predictive value
PR-BSI: PICC-related bloodstream infection
ROC: Receiver operating characteristic
SF2H: French society of infection control
SRLF: French intensive care society
3GCR: 3rd generation cephalosporin resistance
95%CI: 95% confidence interval
